# Referral assessment and patient waiting time decisions in specialized mental healthcare: an exploratory study of early routine collection of PROM (LOVePROM)

**DOI:** 10.1186/s12913-022-08877-4

**Published:** 2022-12-20

**Authors:** Fan Yang, Knut Reidar Wangen, Mattias Victor, Ole André Solbakken, Per Arne Holman

**Affiliations:** 1 Lovisenberg Diaconal Hospital, P.O. box 4970, 0440 Nydalen, Oslo Norway; 2 Department of Health Management and Health Economics, University of Oslo, P.O. box 1089, 0318 Blindern, Oslo Norway; 3 Present Address: Centre for Care Research, Norwegian University of Science and Technology (NTNU), 2802 Gjøvik, Norway; 4 The Department of Psychology, University of Oslo, P.O. box 1094, 0317 Blindern, Oslo, Norway

**Keywords:** Referral assessment, Assessment of healthcare need, Prioritizing, CORE-OM, PROM, Delivery of care, Quality improvement

## Abstract

**Background:**

Norway has prioritized health services according to the principle of “severity of conditions”, where waiting time reflects patients’ medical urgency. We aim to investigate if the “severity-of-condition” principle performs well in the priority setting of waiting time, between and within groups of patients using community mental health services. We also aim to investigate the association between patients’ diagnoses and symptom severity at the start of treatment and the corresponding waiting time.

**Methods:**

The study analyzed routine data from Lovisenberg electronic Patient-Reported Outcome Measurement (LOVePROM) at Lovisenberg Diaconal Hospital in Norway. We estimated patient-reported severity by using Clinical Outcomes in Routine Evaluation – Outcome Measure (CORE-OM), together with patients’ diagnoses to identify patients’ needs in general. To assess the performance of current prioritization, we compared waiting times for patients with major depressive disorder and their maximum recommended waiting time. Multivariate regression models were used to assess the association between patient-reported severity, their diagnosis, and waiting times.

**Results:**

Of the 6108 mental health disorder patients, patients with moderate to severe conditions waited seven weeks, while patients with mild conditions or below clinical cutoff waited 8 weeks. Included in the sample, 1583 were diagnosed with depression. Results indicated that patients with moderate and severe depression had a slightly shorter wait-time than patients with mild depression. However, 32.4% patients with moderate depression and 83.3% patients with severe depression, waited longer than their maximum recommended waiting time. CORE-OM identified depressive patients with risk-to-self harm, who had a 0.84 weeks shorter wait-time. These results were also applied to patients with other common mental health disorders.

**Conclusion:**

Overall, patients waited in accordance with the “severity of condition” principle, but the trend was not strong. Therefore, we advocate that there is substantial room for quality improvements in priority setting on waiting time. We suggest further research should investigate if routine collection of PROM and assessment of referral letters, can better inform specialists when deciding on waiting time.

**Supplementary Information:**

The online version contains supplementary material available at 10.1186/s12913-022-08877-4.

## Background

According to the mental health action plan for 2013–2020 by WHO, the provision of comprehensive, integrated mental health and social care services in community-based settings are essential [1]. If such services are to be affordable and accessible, all countries will have to prioritize when patients’ needs and demands for services exceed providers’ capacity of delivering them. Some patients will have to wait, while others, with lesser needs, may have to accept incomprehensive care. In Norway, referrals can be rejected if the reason for being referred does not fulfill priority-setting criteria [2]. One strategy to secure fairness and effectiveness in accessing affordable health service of good quality can be to apply priority-setting rules and grant patients with legal rights.

A healthcare system without waiting time requires enough capacity, which is probably too expensive to provide [3–5]. Even in the best-balanced healthcare systems, there is some waiting time. Since 1987, Norway has prioritized health services according to the principle of “severity of conditions”, where waiting time should reflect patients’ medical needs. In 1999, the Patients’ Rights Act introduced more specific measures such as waiting time targets and maximum waiting times. These were consistent with other nations such as Finland, Sweden, Denmark, UK and the Netherlands [6]. The Norwegian priority-setting policy has been revised several times [7]. To ensure horizontal equity in medical conditions, from institutional and geographical heterogeneity in praxis and capacity, national priority guidelines were specified for 30 different diagnostic groups in the years 2008–2009 [7]. These guidelines include eight psychiatric diagnoses and 22 somatic diagnoses. For example, patients with severe and moderate depression are recommended to wait no longer than 2 weeks and 8 weeks, respectively. The overall national waiting time target for mental health services in 2021, is 40 days for adults, 35 days for children and adolescents, and 30 days for alcohol and substance dependencies. Detailed description of the Norwegian white papers on priority setting are presented elsewhere [8].

Despite the existence of the Patients’ Rights Act and national generic priority setting guidelines, few studies have examined the performance of these regulations, when it comes to implementing the “severity of conditions” principle. From a Norwegian national-level study, it was reported that although waiting times were reduced after maximum waiting targets were enforced, patients with severe medical conditions were given low-priority [9, 10]. They also reported that high-priority patients had higher probability of waiting excessively. A Norwegian regional-level analysis, found no direct association between actual waiting time and patients’ priority status [11]. They also speculated that implementing overall waiting time guarantees may have pushed the actual waiting time closer to the maximum. Due to the lack of evidence in mental health services, there is a need to investigate whether the “severity of conditions” principle for people with mental illness has worked adequately under the policy.

In Norway, as in many countries, a referral from a general practitioner (GP) is necessary to access specialized healthcare. Several studies report that the specialists who received the referral letter often finds the information insufficient to assess the condition and prioritize the urgency [12–18]. Therefore, disagreements between specialists within and across providing institutions, on what patients’ needs are and who will benefit from specialized treatment, can lead to inequalities in access [19, 20].

Evidence suggests that waiting time is associated with patient outcome [21] and satisfaction [22, 23]. Targeting patients with the greatest needs and letting them have shorter wait-times is at the core of priority setting for elective treatment, similar to triage in acute care. A healthcare system that allows specialists to exercise discretion when assessing individual patients’ needs, must expect substantial variation. A supplement strategy could be to introduce the patients’ subjective opinions on their needs, without taking away the specialist’s ability to make the decisions. Such a strategy appears to be time and resource consuming. However, with the introduction of Patient-Reported Outcomes Measurement Information Systems (PROM-IS) in healthcare, clinicians and clinical managers have the opportunity to include the patient’s self-assessment in the decision-making process and planning of care. Pioneers of PROM-IS, e.g., New England Baptist Hospital (NEBH), have invested substantially in strategies that aim to provide patients with greater influence over their own care. For instance, based on Patient-Reported Outcomes Measurement (PROM) collection at NEBH, a predictive AI model [24] measures potential risks before treatment and is used to share decision making between patients and providers.

Studies of referral texts and specialists’ clinical assessment in electronic medical records (EMR) require extensive resources and patient consent. The Lovisenberg electronic Patient Reported Outcome Measurement (LOVePROM) project, collects PROM as part of a quality register at a community mental health center (CMHC) in Oslo. The project was able to provide an anonymous dataset of administrative data from the electronic medical records, with waiting time, codes from the 10th revision of the International Statistical Classification of Diseases and Related Health Problems (ICD-10) and PROM, episodes, and procedures. In this case, the content of the referral letter is unknown. Without the GPs’ description of urgency and need, historic waiting times should work as a proxy to urgency and needs, as perceived by the clinicians at Lovisenberg hospital.

To create groups of patients, this study investigates ICD-10 diagnosis, particularly regarding severity in depression. As noted, Norwegian clinical guidelines have specific recommendations for waiting time in depression. To examine individual needs, we use PROM collected just before the onset of therapy, specifically data from the Clinical Outcomes in Routine Evaluation-Outcome Measure (CORE-OM).

### Aim of the study

This study investigates if patients’ prioritization on waiting time is consistent with the principle of “severity of conditions”, where waiting time should reflect patients’ medical urgency within the given capacity. Specifically, we examine the association between patients’ diagnoses and symptom severity at the start of treatment and their waiting time. If the system works well, we hypothesize that patients reporting severe distress have had significantly shorter wait-times, compared with low and moderately distressed groups measured by CORE-OM. We also hypothesize that severely depressed patients have had shorter wait-times by several weeks than patients with moderate to mild depression. The findings will be discussed in relation to when pre-PROM should be collected— together with referral assessment or at onset of treatment after waiting time. This quality improvement study is significant as the findings will help highlight if there are good reasons to recommend a shift from PROM collection after waiting time is over, to before waiting time is set.

## Method

### Materials

This study analyzed routinely collected data from EMR and PROMs at Lovisenberg Diaconal Hospital CMHC in Oslo. The CMHC covers a catchment area of approximately 200,000 inhabitants in Oslo’s inner city, with five clinics providing services for mental disorders, addiction, and substance use disorders. One of these clinics is defined as a return-to-work program, where also mild conditions could be prioritized to prevent workers from sick leave. We included new patients referred to specialized mental health outpatient programs between January 2017 and August 2020.

**Fig. 1:**
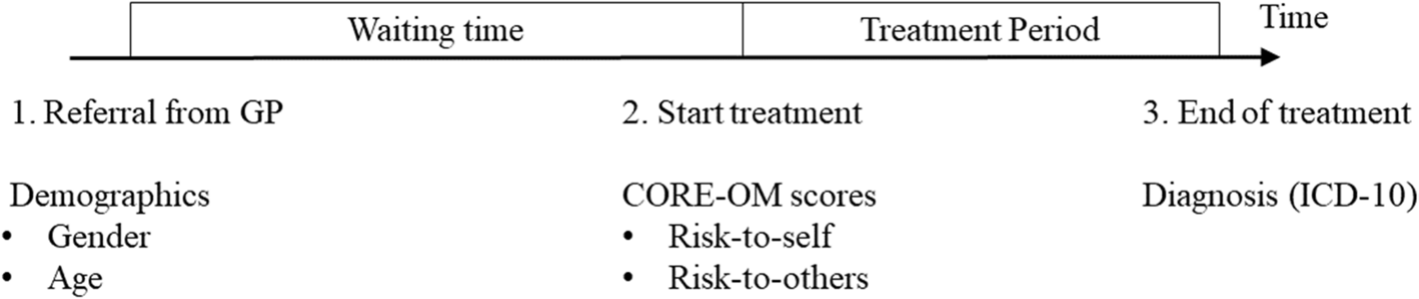
Timeline for referral, waiting time and treatment (with data collection timepoints)

The data set was extracted from Lovisenberg CMHC’s quality register (LOVePROM) in an anonymous form. Using CORE-OM, each patient’s health status was assessed three days before the first consultation. The CORE-OM (Norwegian version^1^) is a 34-item global distress measure with four subscales: subjective well-being, problems/symptoms, life functioning, and risk/harm [25]. Patients’ final diagnoses were received by the EMR at the end of treatment. Figure 1 summarizes the data collection time-points and the main information utilized.

### Variables

Table 1 presents a list of the variables used. Waiting time was defined as the time between the referral from the GP and the start of treatment (Fig. 1) and was measured in weeks. In our regression analyses, waiting time was the dependent variable while the other variables were independent.

The health need characteristics were collected at the start of treatment using the CORE-OM questionnaire. The CORE-OM total score was based on all 34 questions in the instrument. The score is defined on an ordinal scale ranging from 0 to 4 for each question and 0–136 for the 34 questions in total. We used the mean score for all questions multiplied with 10, so that CORE-OM total score ranges between 0 and 40. In parts of our statistical analysis, we categorized the CORE-OM total score into five CORE-OM severity categories [26, 27]: Under clinical cut-off (0–10); Mild (10-15); Moderate (15-20); Moderate severe (20-25); and Severe (25–40).

The dichotomous variable of being at “risk-to-others” was based on two risk questions (Q6 and Q22) from the CORE-OM questionnaire, confer Supplementary Table 1, Additional File [25, 26, 28]. If a participant responded to at least one of the questions that was above a given trigger level, the risk variable was assigned the value 1, otherwise the risk variable took the value 0 (i.e., if the responses to both risk questions were below the trigger levels). The variable of being at “risk-to-self” was similarly defined based on a set of four risk questions (see Supplementary Table 1, Additional File).

The diagnosis variables (ICD-10) were recorded at the last episode of treatment for the main categories: Mental and behavioral disorders (F-chapter), Symptoms (R-chapter), and Factors influencing health status (Z-chapter). For patients with depression, we also obtained variables for eight diagnostic subcategories.

### Data analysis

The original sample consisted of 6314 patient records. After excluding 206 incomplete records, the net sample consisted of 6108 patients. From this sample, we defined a subsample consisting of 1583 patients with depression.

We estimated basic descriptive statistics for the included variables— means and standard deviation for the continuous variables, and sample proportions for the dichotomous variables.

For patients with depression, the national guidelines advise maximum waiting times by level of severity (2 weeks for severe depression; 8 weeks for moderate depression). For our subsample of patients with depression, we constructed box plots for the observed waiting time by the severity level. This enabled us to compare the distribution of waiting times with the national guidelines.

For mental health patients in general, the national political target for the average waiting time was to be under 40 days in the year 2020 (approximately 6 weeks). Except for depression, as mentioned, the guidelines do not state maximum waiting times depending on ICD codes. To assess the association between waiting time and severity measured at the start of the treatment, we constructed box plots for the waiting time based on CORE-OM severity categories.

Waiting times can potentially be associated by any of our included variables and the associations can potentially confound each other. To investigate this, we estimated four linear regression models (Model I–IV) for the sample of patients with depression, using waiting time as the dependent variable and different sets of independent variables.

In Model I, we included gender and age – variables that were available at the time of referral. In Models II and III, we added the health status characteristics (CORE-OM available at the start of treatment) and the depression diagnosis variables (available at the end of treatment), respectively. Model IV included both health status characteristics and the depression diagnosis variables. It can be used to assess whether these two sets of variables confound each other or compete for explanatory power. Explanatory power was measured by the adjusted coefficient of determination.

We then estimated corresponding regression models for the full sample consisting of all patients (Models V–VIII). The difference between Models I–IV and these models is that the diagnosis variables in the latter were based on ICD-10 main categories, while in the former, were based on ICD-10 (depression) subcategories. We used Stata 16.0 to perform the analyses and a 10% significance level.

## Results

Table 1 shows the descriptive statistics for the sample and variables included in the regression models. Our study sample was predominantly female, 20–39 years old, and diagnosed with Mood [affective] disorders, Neurotic, stress-related, and somatoform disorders. Patients with depression (*n* = 1583) accounted for 25.8% of the whole sample. The mean CORE-OM score was 17.4, with a standard deviation of 6.0 scores, that is, over the clinical cut-off being 10. The distribution of patients according to CORE-OM categories are reported in Supplementary Table 2, Additional File. Very few patients (1.1%) reported being of risk to others, while a much larger group (19.0%) reported being at risk of self-harm. During the study period, patients in the study sample waited, on average, 8.1 weeks before treatment, varied by 4.9 weeks (Table 1).

**Table 1:** Descriptive statistics for the sample used in the regression analyses (*N* = 6108)^a)^

Variable	Proportion in %	Mean (Std. Dev.)
**Waiting time (weeks)**		8.1(4.9)
**Patient demographics**		
Male	31.5	
Age < 19	1.8	
Age 20–29	36.1	
Age 30–39	34.1	
Age 40–49	17.1	
Age 50–59	8.8	
Age > 60	2.2	
**Health need characteristics (patient reported outcome measures)**		
CORE-OM total scores^b^		17.4(6.0)
Risk-to-others	1.1	
Risk-to-self	19.0	
**ICD-10 Main Category**		
F00-F09: Organic, including symptomatic, mental disorders	0.0	
F10-F19: Mental and behavioral disorders due to psychoactive substance use	3.7	
F20-F29: Schizophrenia, schizotypal and delusional disorders)	0.8	
F30-F39: Mood [affective] disorders	32.9	
F40-F49: Neurotic, stress-related and somatoform disorders	44.5	
F50-F59: Behavioral syndromes associated with physiological disturbances and physical factors	6.2	
F60-F69: Disorders of adult personality and behavior	3.4	
F80-F89: Disorders of psychological development	0.4	
F90-F99: Behavioral and emotional disorders with onset usually occurring in childhood and adolescence	3.6	
R00-R99: Symptoms, signs and abnormal clinical and laboratory findings, not elsewhere classified	4.1	
Z00-Z99: Factors influencing health status and contact with health services	0.5	
**ICD-10 Subcategory for depression (25.8%)** ^c^ Mild depression (8.4%)		
F32.0 Major depressive disorder, single episode, mild	5.6	
F33.0 Major depressive disorder, recurrent, mild	2.8	
Moderate depression (16.3%)		
F32.1 Major depressive disorder, single episode, moderate	9.8	
F33.1 Major depressive disorder, recurrent, moderate	6.5	
Severe depression (1.1%)		
F32.2 Major depressive disorder, single episode, severe without psychotic features	0.6	
F32.3 Major depressive disorder, single episode, severe with psychotic features	0.0	
F33.2 Major depressive disorder, recurrent severe without psychotic features	0.5	
F33.3 Major depressive disorder, recurrent, severe with psychotic symptoms	0.0	

^a^Most variables are dichotomous, and the mean represent the percentages of patients with the characteristic in the leftmost column. ^b^CORE-OM scores range from 0 to 40, where 10 is defined as clinical cut-off. ^c^Depression diagnosis: (1) Mild depression (*N* = 513): F32.0 Major depressive disorder, single episode, mild, F33.0 Major depressive disorder, recurrent, mild; (2) Moderate depression (*N* = 998): F32.1 Major depressive disorder, single episode, moderate, F33.1 Major depressive disorder, recurrent, moderate; (3) Severe depression (*N* = 72): F32.2 Major depressive disorder, single episode, severe without psychotic features, F32.3 Major depressive disorder, single episode, severe with psychotic features; F33.2 Major depressive disorder, recurrent severe without psychotic features; F33.3 Major depressive disorder, recurrent, severe with psychotic symptoms

**Fig. 2:**
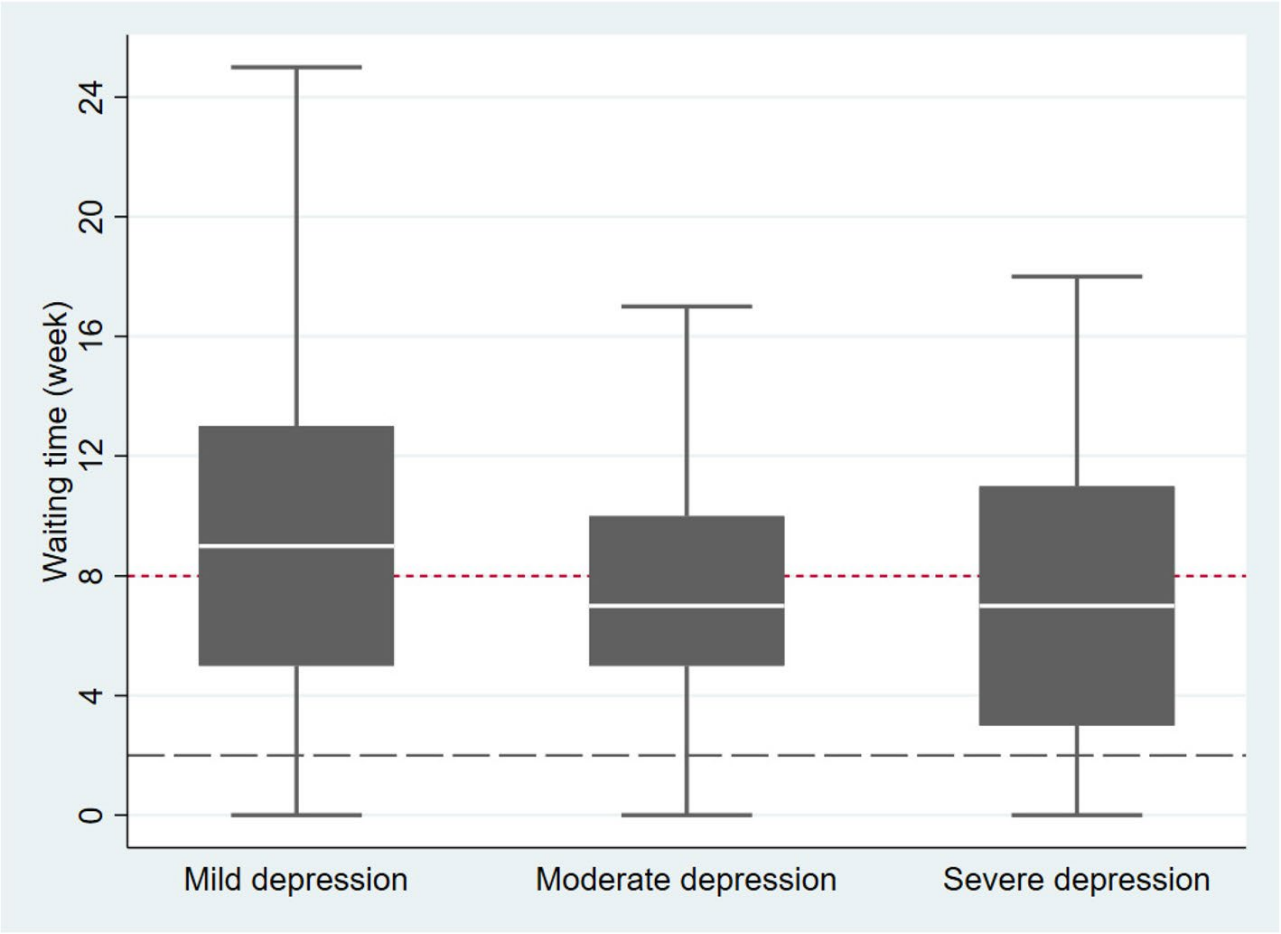
Waiting time for patients with depressive disorders, by depression diagnosis (*N* = 1583) Note: The distribution of waiting time for three categories of depression were significantly different (Kruskal-Wallis test with p-value < 0.001). The lower and upper dashed lines represent the Norwegian national guidelines’ maximum waiting time for severe depressive disorder (2 weeks) and moderate depressive disorder (8 weeks), respectively [29]

**Fig. 3:**
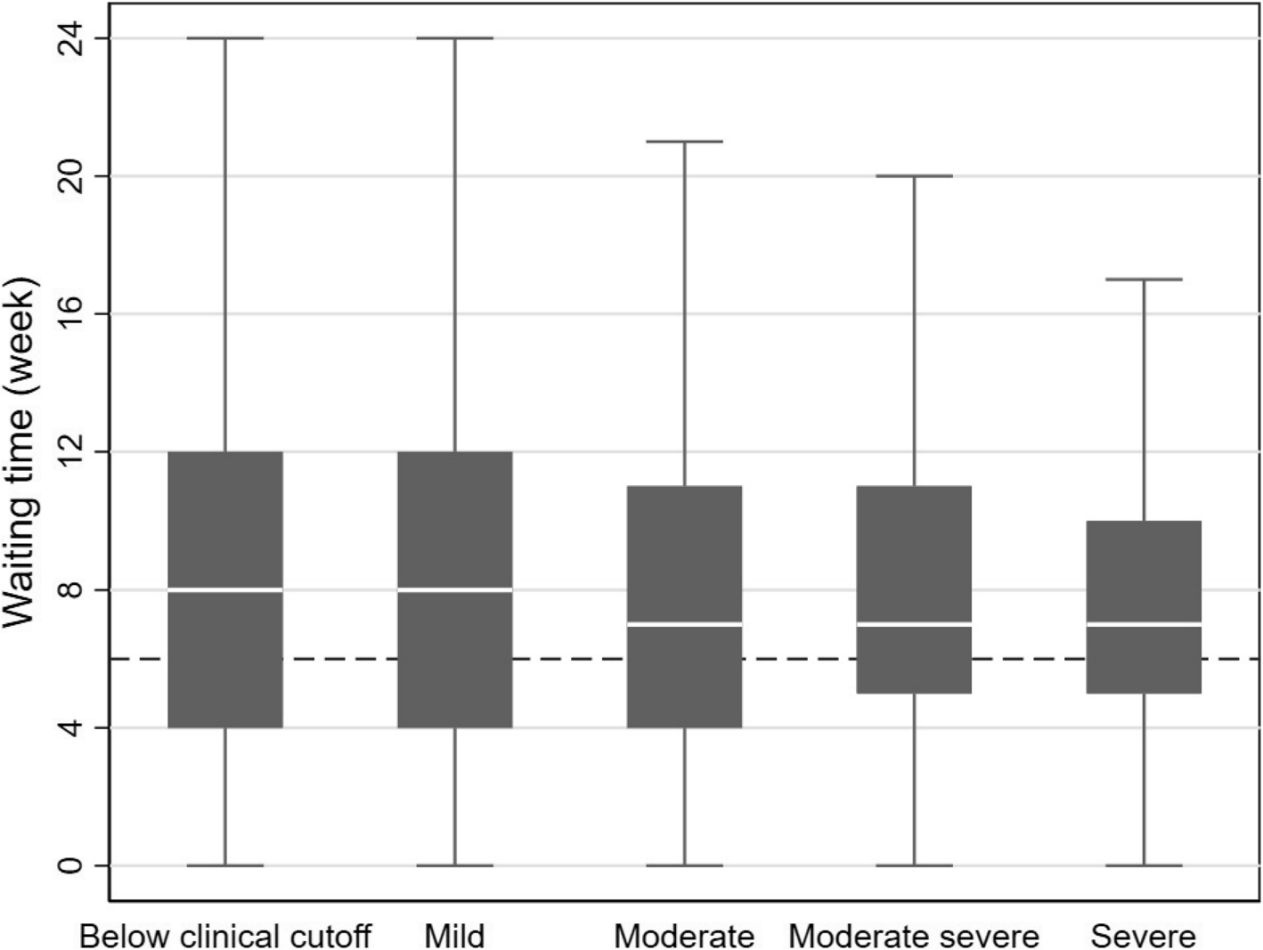
Waiting time by CORE-OM severity category (*N* = 6108) Note: The dashed line at approximately 6 weeks (40 days) represents the Norwegian national target for average waiting time for mental health outpatients [8]

The results of the prioritization performance are presented using boxplots in Figs. 2 and 3. In the sample, waiting time varied from a median of 7 weeks for patients with severe and moderate depression, to 9 weeks for those with mild depression (*p* = 0.000). The boxplot depicts overlap among the three severity levels and variation within each level. The results also demonstrated that waiting times substantially exceeded national guideline recommendations. Patients with moderate depression (32.4%) and severe depression (83.3%) waited longer than 8 weeks and 2 weeks, respectively (Fig. 2).

The results for overall diagnoses also showed the waiting time exceeding the national prioritization target (dashed line: 6 weeks). The variance narrows as the severity increases, while medium waiting times do not indicate a steady decrease. Specifically, patients with moderate to severe conditions waited seven weeks, while patients with mild conditions or below clinical cutoff waited 8 weeks. The difference was significant but small (Cohen’s d = 0.198, *p* = 0.069)  (Fig. 3).

The multivariate regression models used information obtained at different time points to elaborate potential factors that correlated with waiting time (see Table 2). Model I shows that patients over 60-year-old wait 1.8 weeks longer than those aged 20–29 years. Longer wait-times in this group can be found after including more information in Model II and Model III.

Model II adds on the variables’ CORE-OM scores, risk-to-others, and risk-to-self. As CORE-OM scores rise by one point, the coefficient of CORE-OM denotes the difference in waiting time. In Model II, the estimated value is -0.069, which means that an increase of one unit on the CORE-OM would reduce waiting time by less than one day. It shows a significant, but small impact (Cohen’s d = 0.141)^2^. The coefficients for risk-to-self and risk-to-others denote the change in waiting time when comparing patients’ CORE-OM risk subgroups above and below the risk-alert line. The significant estimate for risk-to-self was -0.906 (*p* < 0.01) for Model II and -0.844 (*p* < 0.01) for Model IV, showing that the patients with the risk-to-self alert waited around 1-week less than those without.

Models III and IV show regressions that include a depression diagnosis, while Model III excludes CORE-OM. Compared with patients with mild depression, patients with moderate and severe depression have shorter wait-times (Model III & Model IV).

Measured by the magnitude, with PROM estimates in Model IV, the moderately and severely depressed patients had shorter wait-times by 1.3 weeks (Cohen’s d = 0.26) and 1.5 weeks (Cohen’s d = 0.29), respectively. This magnitude is slightly smaller than estimates without PROM, which are shorter by 1.5 and 2.1 weeks (Model III).

Results from Model II and Model IV show that CORE-OM scores and depression stages are competing for explanation power. The change of CORE-OM scores is consistent with the change in depression stages. As we included the depression diagnosis, the CORE-OM scores decreased from significant (coefficient = -0.069 in Model II) to insignificant (coefficient = -0.027 in Model IV). The unit change from moderate to severe depression yields an increase of 4.8 CORE-OM scores.

**Table 2:** Multivariate regression models for waiting time. Patients with depressive disorders (*N* = 1583)^a^

	Model I		Model II		Model III		Model IV	
	Coeff	Std.err	Coeff	Std.err	Coeff	Std.err	Coeff	Std.err
Male	0.021	0.257	0.142	0.256	0.150	0.255	0.213	0.255
*Age groups*								
< 20	-1.480	1.067	-1.240	1.059	-1.405	1.054	-1.295	1.052
20–29 (ref)	-	-	-	-	-	-	-	-
30–39	0.364	0.281	0.095	0.285	0.235	0.278	0.052	0.283
40–49	-0.154	0.351	-0.446	0.352	-0.271	0.347	-0.458	0.350
50–59	0.383	0.536	0.220	0.536	0.258	0.442	0.121	0.443
> 60	**1.817**	0.768	**1.429**	0.765	**1.430**	0.762	1.225	0.762
CORE-OM scores			**-0.069**	0.026			-0.027	0.028
Risk-to-self			**-0.906**	0.325			**-0.844**	0.324
Risk-to-others			0.393	1.425			0.366	1.417
*Depression diagnosis* ^b)^								
Mild depression (ref)					**-**	**-**	**-**	-
Moderate depression					**-1.547**	0.253	**-1.280**	0.271
Severe depression					**-2.050**	0.584	**-1.483**	0.609
Constant	**7.805**	0.382	**9.362**	0.594	**8.786**	0.408	**9.357**	0.591
Adj R-sq	0.0029		0.020		0.027		0.033	

^a^Coefficients with two-sided p-values less than 10% are in boldface

Diagnoses do not directly describe the actual severity of conditions. Therefore, we used PROM as an expression of patient severity. Table 3 reports the waiting time and influential factors for all patients with ICD-10 diagnosis. Compared with the reference group (aged 20–29), the youngest patients (age < 20) had significantly shorter wait-times— nearly one week less (-0.917; Model V). After controlling for severity in CORE-OM scores in Model VI, we observed that older patients (aged 40–49 and 50–59) have somewhat shorter wait-times.

**Table 3:** Multivariate regression models for waiting time. Coefficients and standard errors (*N* = 6108)^a^

	Model V		Model VI		Model VII		Model VIII	
	Coeff	Std.err	Coeff	Std.err	Coeff	Std.err	Coeff	Std.err
Male	-0.141	0.136	-0.106	0.136	-0.163	0.135	-0.146	0.135
*Age groups*								
< 20	**-0.917**	0.483	-0.780	0.483	-1.115	0.475	**-0.979**	0.475
20–29 (ref)	-	-	-	-			-	-
30–39	0.142	0.151	0.022	0.152	0.164	0.149	0.072	0.150
40–49	-0.244	0.186	**-0.376**	0.187	-0.219	0.183	**-0.315**	0.184
50–59	-0.363	0.237	**-0.469**	0.238	-0.216	0.234	-0.287	0.235
> 60	0.156	0.441	-0.021	0.442	0.431	0.435	0.301	0.435
CORE-OM scores			-0.016	0.013			**-0.023**	0.013
Risk-to-self			**-0.743**	0.194			**-0.538**	0.192
Risk-to-others			0.580	0.602			0.757	0.592
*ICD-10 categories* ^b^								
F00 – F09					**5.787**	3.484	**5.961**	3.479
F10 – F19					**1.435**	0.762	**1.516**	0.761
F20 – F29 (ref)					-	-	-	-
F30 – F39					**3.258**	0.699	**3.310**	0.699
F40 – F49					**3.640**	0.697	**3.566**	0.697
F50 – F59					**1.374**	0.735	**1.237**	0.735
F60 – F69					**2.813**	0.769	**3.022**	0.770
F80 – F89					**5.552**	1.203	**5.584**	1.203
F90 – F99					**6.638**	0.762	**6.541**	0.762
R00 – R99					**3.409**	0.756	**3.282**	0.756
Z00 – Z99					**6.216**	1.134	**6.062**	1.133
Constant	8.312	0.202	8.749	0.293	4.958	0.722	5.503	0.746
Ad R-sq	0.0009		0.0053		0.039		0.0422	

^a^Coefficients with two-sided p-values less than 10% are in boldface

^b^ICD–10 diagnosis: F00–F09: Organic, including symptomatic, mental disorders; F10–F19: Mental and behavioral disorders due to psychoactive substance use; F20–F29: Schizophrenia, schizotypal and delusional disorders; F30–F39: Mood [affective] disorders; F40–F49: Neurotic, stress–related and somatoform disorders; F50–F59: Behavioral syndromes associated with physiological disturbances and physical factors; F60–F69: Disorders of adult personality and behavior; F80–F89: Disorders of psychological development; F90–F99: Behavioral and emotional disorders with onset usually occurring in childhood and adolescence; R00–R99: Symptoms, signs and abnormal clinical and laboratory findings, not elsewhere classified; Z00–Z99: Factors influencing health status and contact with health services

Compared with patients without a risk-to-self alert, patients with an alert had a shorter wait-time by 0.7 weeks in Model VI and by 0.5 weeks in Model VIII. The coefficient for CORE-OM scores in Model VIII indicates that patients with a one-unit increase in CORE-OM had nearly 0 (0.023 ; Model VIII; *p* < 0.1)-week shorter waiting time. Model VII was analyzed without PROM. Comparing with PROM in Model VIII, the increase of adjusted R squared changed from 3.9 to 4.2%. Further, patients in different diagnostic groups had systematically different waiting times. All other diagnostic groups waited longer than the reference group F20–F29 (Schizophrenia, schizotypal and delusional disorders). The effect was largest for patients diagnosed with F90–F99, waiting 6.5 and 6.6 weeks longer than the reference group with or without PROM data (Model VIII & Model VII) (Table 3).

## Discussion

In this exploratory study, we found that diagnoses and symptom severity, described by PROM at the start of treatment, were associated with waiting time. However, the associations were smaller than expected, and many patients waited longer than the recommendations of the official guidelines.

In this study, the overall mean waiting time was eight weeks, two weeks longer than the national target in 2021. This could be because of limited capacity in the services. Compared with patients with schizophrenia, other diagnostic groups had longer wait-times. This is consistent with clinical recommendations. Relatively few patients were diagnosed with an unspecific diagnosis (R00–R99 and Z00–Z99). This indicates that patients with mental health issues primarily requiring specialized treatment were accepted into the health service. However, a more specific referral evaluation is still required, as indicated by a relatively high proportion of patients with mild depression diagnosis. We found a significant association with shorter waiting time for the youngest patients, but it was less than a week.

The patients reported a mean CORE-OM total score of 17.4, with a standard deviation of 6.0. This indicates that the CMHC prioritize patients that are over the clinical cut-off of 10. In this respect, referral letters seem to inform the priority setting well. There was an association between higher total scores on CORE-OM and shorter waiting times, but this association was weak. A ten-point higher score on CORE-OM, which is a clinically relevant size, was associated with only 0.2 weeks, i.e., one-two days shorter waiting time (Table 3, Model VIII).

The most prominent finding of our study is that severely depressed patients wait much longer than recommended. Nearly all patients diagnosed with severe depression waited longer than the recommended two weeks. This might indicate a shortcoming in referrals if they do not identify the most severe cases within the most frequent mental disorder. Most patients reporting moderate-severe and severe levels of distress on the generic instrument CORE-OM waited five to ten-eleven weeks. An alternative explanation is that some of these patients’ condition became worse during the relatively long waiting time.

Patients with depressive disorders, reporting risk to self-harm and patients with moderate to severe depression had a shorter wait-time by less than two weeks, than patients without self-harm alert and mild depression, when gender, age, and CORE-OM scores were adjusted for (Table 2, Mode IV). This is consistent with our observations (Fig. 2) where patients with generally higher symptom severity waited almost as long as patients with milder symptoms, possibly indicating failure of referrals to identify more severe conditions. Compared to diagnosis, PROM identifies patients being at risk-to-self and providing them with shorter waiting times (Table 2, Model II, IV & Table 3, Model VI, VIII).

The assessment of risk is a top priority within routine counselling and psychotherapy services. In recent years, staff have received training in this area. The risk domain of CORE-OM, alert clinicians to patient’s risk of harm to self and others. Responses on risk domains do not have a high predictive value. However, the alert gives providers an opportunity to consider treatment actions to relieve distress and potentially prevent harm, e.g., shorter waiting time. Research suggests that differences between practitioner-rated and client self-report assessments are to be expected and has indicated that the rates of difference can be relatively high (i.e., > 50%). Bewick and colleagues found that the CORE-OM risk domain identified 44% of clients as “at risk” while the practitioner assessment identified 10% of clients as being “at risk” [30]. For the overall sample in their study (*n* = 25338), 18% of clients were classified by the practitioner as presenting no risk when the CORE-OM risk domain identified them at risk [30].

In our study, relatively few (1%) patients reported a violence alert and a relatively large group of patients (19%) reported elevated risk of harm to self. Their wait-times were similar (risk-to-others) or were shorter by less than one week (risk-to-self), compared with patients reporting under the thresholds for these risk behaviors. This observation could be viewed as consistent with Bewick and colleagues reporting that clients and practitioners have different views of risk. The difference in the size of patients identified at risk— 44% reported by their study and 19% reported in our study— can result from different patient selections or thresholds [30].

The most comprehensive model in our study including all factors (Table 3, model VIII), explained only 4.2% of the variation in waiting time. This indicates that factors other than diagnoses and symptom severity at onset of treatment, explain most of the variation in waiting time. These factors may be: long waiting time may have worsened the condition from time of referral to onset of treatment; information on risk behavior might not have been described by GP’s, and therefore, not available when referrals were assessed; GP’s may not have asked patients risk questions, or patients may not have trusted their GPs with their honest answers. Lack of trust in GPs and lack of openness with their current mental health state, could be one reason why patients ask for a referral to specialized mental health services. Lack of competence or time to deal with mental health issues could be GPs’ reason to refer. Moreover, clinicians evaluating referrals may give weight to factors other than condition severity, for example, information about earlier treatment, motivation, or psychosocial factors.

Variation in waiting time normally expresses prioritization consistent with the principles of horizontal and vertical equity. The World Health Organization in the year 2000 advocates concepts of horizontal equity— individuals or groups with the same level of need are treated equally [31]. Vertical equity relates to cases where people with unequal needs are treated proportionately differently, preferentially providing to those with the greatest need.

Previous studies have found that quality of referrals and other forms of communications in transition of care can affect patient’s safety, e.g., when urgency is not described or inadequately understood by providers in the pathway [32]. Holman and colleagues described how disagreement on referral assessment may lead to random priority setting effects in a study setting [20]. Twenty anonymous case vignettes referrals were classified by 42 admission team members at 16 CMHCs. The same “patient” was assessed as in urgent need or no need at all, suggesting low degree of interrater reliability. A way of improving quality in assessment of referrals could be to have all referred patients undergo an evaluation by a specialist as part of the priority setting process.

A more cost-efficient alternative could be to collect PROM with a validated measurement at the time of referral, allowing the patients’ own descriptions of health condition and symptom severity to inform the intake process.

### Recommendations and limitations

To our knowledge, PROM is not routinely used anywhere to accompany the assessment of referral letters for priority-setting decisions. We suggest that this is implemented, and that further research investigates possible effects on priority setting and waiting time compared to guidelines.

There are also some limitations in the setting. First, there is inconformity of data collection timepoints. Ideally, we would wish to know patients’ subjective health status when the referral letter is evaluated and waiting time is set. In this study, such data was not available to inform decision making on waiting time together with referral assessment. Therefore, we are not looking for agreement, as for inter-rater reliability, between specialists at CMHC and patients perceived the urgency. They did not share the same information at the time. Second, the GPs problem description (International Classification of Primary Care codes) and ICD-10 codes are not the same, and patient’s condition described by PROM may well have changed during waiting time and treatment, resulting in a different ICD-10 classification compared with the first consultation. We only have ICD-10 codes and find that the last code best represents the diagnostic conclusion. This is a limitation of the study setting. Our aim was to determine whether the current practice of referral assessment works well in accordance with the priority setting principal of “severity of the condition”. Since PROM is not routinely used anywhere to accompany the assessment of referral letters for priority-setting decisions, we have not yet been able to perform a naturalistic study. With LOVePROM or other electronic infrastructure for administering questionnaires to patients, it could be feasible to assess CORE-OM from patients together with referral letter within days of the referral. Third, the risk-to-self and risk-to-others items are dependent on our clinical therapeutic exercise, which may or may not apply to other clinical settings in mental health clinics.

## Conclusions

Our results have direct implications for the waiting time target policy, and routine collected outcome measures. As expected, this study reports variation in waiting time between groups of patients at a CMHC. Overall, the patients wait in accordance with the *severity of condition* principle, i.e., higher urgency leads to shorter waiting times. However, the trend is not strong. Therefore, we advocate that there is substantial room for quality improvements in priority setting on waiting time. The role of managing access to specialized health services should aim to target patients with high burden of disease and with risk of reduced outcomes if waiting time is too long [11, 33, 34].

Without validated rating instruments and checklists, the room to exercise clinical discretion is large. PROM, in this case CORE-OM, has some explanatory power. We suggest it could be substantially stronger if PROM could inform decision makers before setting waiting time.

Based on these examples and our observations, we argue that waiting time targets, for groups of patients, not only depend on capacity. Such policy’s efficiency, i.e., the principle of severity of condition or the principles of horizontal and vertical equity, depend on an agreement on which patients belongs in the prioritized groups. Therefore, we suggest that future studies should investigate if routine collection of pre-PROM, assessed together with referral letters, can better inform specialists when deciding on waiting time. We expect to find that such a reform will improve equity, so that patients with higher distress and risk of lost prognosis, have shorter wait-times. We also expect to find less variation in waiting time within groups of similar conditions. We encourage the LOVePROM project to start collecting pre-PROM and evaluate the effect on priority setting.

## Supplementary Information

**Additional file 1:** **Table 1.** Trigger levels of the Risk-to-others and Risk-to-self, based onquestions from the CORE-OM questionnaire. **Table 2.** The distribution of patients by CORE-OM categories.

## Data Availability

The data that support the findings of this study are available from Lovisenberg Diaconal Hospital but restrictions apply to the availability of these data, which were used under license for the current study, and so are not publicly available. Data are however available from PAH upon reasonable request and with permission of Lovisenberg Diaconal Hospital.

## Abbreviations

PROM: Patient-Reported Outcomes Measurement
GP: General practitioner
PROM-IS: Patient-Reported Outcomes Measurement Information System
NEBH: New England Baptist Hospital
ICD-10: The 10th revision of the International Statistical Classification of Diseases and Related Health Problems
EMR: Electronic medical records
LOVePROM: Lovisenberg electronic Patient Reported Outcome Measurement
CMHC: Community mental health center
CORE-OM: Clinical Outcomes in Routine Evaluation-Outcome Measure

## Footnotes

1 See Skre et al. (2013).The Norwegian version of CORE-OM is validated by this research [28].

2 Cohens d. 10 points up on CORE = -0.69 weeks divided by the SD of 4.9 weeks yields a Cohen’s d = 0.141 (insubstantial).

## Declarations

privacy office (reference no. 2021/1739) approved use of data for the study without specific written consent from patients, as such data are routinely collected in the context of mandatory quality assurance. Beyond this, the study was conducted accordance with the relevant.

guidelines and regulations of the Helsinki Declaration.

## References

[1] World Health Organization. Mental health action plan 2013–2020 [Internet]. Geneva,Switzerland; 2013. Available from: https://www.who.int/publications/i/item/9789241506021.

[2] Helse- og omsorgsdepartementet. Sosial- og helse departementet: Forskrift om prioritering av helsetjenester, rett til nødvendig helsehjelp fra spesialisthelsetjenesten, rett til behandling i utlandet og om klagenemnd [Internet]. Available from: http://lovdata.no/dokument/SF/forskrift/2000-12-01-1208.

[3] Gravelle H, Schroyen F. Optimal hospital payment rules under rationing by waiting. J Health Econ [Internet]. 2020;70:102277. Available from: https://www.sciencedirect.com/science/article/pii/S0167629619302681. 10.1016/j.jhealeco.2019.102277 31932037

[4] Madeira A, Moutinho V, Fuinhas JA. Does waiting times decrease or increase operational costs in short and long-term? Evidence from Portuguese public hospitals. Eur J Heal Econ [Internet]. 2021;22(8):1195–216. Available from: 10.1007/s10198-021-01331-y. 10.1007/s10198-021-01331-y 34106363

[5] Siciliani L, Stanciole A, Jacobs R. Do waiting times reduce hospital costs? J Health Econ [Internet]. 2009;28(4):771–80. Available from: https://www.sciencedirect.com/science/article/pii/S016762960900040X. 10.1016/j.jhealeco.2009.04.002 19446901

[6] Siciliani L, Hurst J (2005). Tackling excessive waiting times for elective surgery: a comparative analysis of policies in 12 OECD countries. Health Policy.

[7] Ringard Å, Sagan A, Sperre Saunes I, Lindahl AK (2013). Norway: health system review. Health Syst Transit.

[8] Helse og omsorgsdepartementet. Styringsmål 2021 [Internet]. 2021 [cited 2022 Mar 3]. Available from: https://www.regjeringen.no/no/tema/helse-og-omsorg/sykehus/styringsdokumenter1/oppdragsdokument/id535564/.

[9] Askildsen JE, Holmås TH, Kaarboe O (2010). Prioritization and patients’ rights: analysing the effect of a reform in the norwegian hospital sector. Soc Sci Med.

[10] Askildsen JE, Holmås TH, Kaarboe O (2011). Monitoring prioritisation in the public health-care sector by use of medical guidelines. The case of Norway. Health Econ.

[11] Gangstøe JJ, Heggestad T, Norheim OF. Norwegian Priority Setting in Practice - an Analysis of Waiting Time Patterns Across Medical Disciplines. Int J Heal policy Manag [Internet]. 2016 Mar 2;5(6):373–8. Available from: https://pubmed.ncbi.nlm.nih.gov/27285515. 10.15171/ijhpm.2016.23 PMC4885728 27285515

[12] Hartveit M, Vanhaecht K, Thorsen O, Biringer E, Haug K, Aslaksen A. Quality indicators for the referral process from primary to specialised mental health care: an explorative study in accordance with the RAND appropriateness method. BMC Health Serv Res [Internet]. 2017 Jan 3;17(1):4. Available from: https://pubmed.ncbi.nlm.nih.gov/28049470. 10.1186/s12913-016-1941-1 PMC5209847 28049470

[13] Grimshaw JM, Winkens RA, Shirran L, Cunningham C, Mayhew A, Thomas R, Fraser C. Interventions to improve outpatient referrals from primary care to secondary care. Cochrane Database Syst Rev. 2005;(3):CD005471. 10.1002/14651858.CD005471. 10.1002/14651858.CD005471 16034981

[14] Durbin J, Barnsley J, Finlayson B, Jaakkimainen L, Lin E, Berta W (2012). Quality of communication between primary health care and mental health care: an examination of referral and discharge letters. J Behav Health Serv Res.

[15] Jiwa M, Dhaliwal S (2012). Referral writer: preliminary evidence for the value of comprehensive referral letters. Qual Prim Care.

[16] Shaw I, Smith KMC, Middleton H, Woodward L. A Letter of Consequence: Referral Letters From General Practitioners to Secondary Mental Health Services. Qual Health Res [Internet]. 2005 Jan 1;15(1):116–28. Available from: 10.1177/1049732304270725. 10.1177/1049732304270725 15574719

[17] Tobin-Schnittger P, O’Doherty J, O’Connor R, O’Regan A (2018). Improving quality of referral letters from primary to secondary care: a literature review and discussion paper. Prim Health Care Res Dev..

[18] Wangen KR, Grepperud S. Supply factors as determinants of treatment costs: clinicians’ assessments of a given set of referrals to community mental health centers in Norway. BMC Health Serv Res [Internet]. 2018;18(1):60. Available from:10.1186/s12913-018-2884-5. 10.1186/s12913-018-2884-5 PMC5789684 29378666

[19] Grepperud S, Holman PA, Wangen KR (2014). Factors explaining priority setting at community mental health centres: a quantitative analysis of referral assessments. BMC Health Serv Res.

[20] Holman PA, Ruud T, Grepperud S. Horizontal equity and mental health care: a study of priority ratings by clinicians and teams at outpatient clinics. BMC Health Serv Res [Internet]. 2012;12(1):162. Available from: 10.1186/1472-6963-12-162. 10.1186/1472-6963-12-162 PMC3430583 22704131

[21] Reichert A, Jacobs R (2018). The impact of waiting time on patient outcomes: evidence from early intervention in psychosis services in England. Heal Econ.

[22] Bjertnaes OA, Garratt A, Iversen H, Ruud T (2009). The association between GP and patient ratings of quality of care at outpatient clinics. Fam Pract.

[23] Desta H, Berhe T, Hintsa S. Assessment of patients' satisfaction and associated factors among outpatients received mental health services at public hospitals of Mekelle Town, northern Ethiopia. Int J Ment Health Syst. 2018;12:38. 10.1186/s13033-018-0217-z. 10.1186/s13033-018-0217-z PMC6042256 30008801

[24] Cyft. Turning Data into Better Surgical Care [Internet]. 2019. Available from: https://www.cyft.com/2019/10/23/cyft-turning-data-into-better-surgical-care/.

[25] Evans C, Connell J, Barkham M, Margison F, McGrath G, Mellor-Clark J, et al. Towards a standardised brief outcome measure: Psychometric properties and utility of the CORE–OM. Br J Psychiatry [Internet]. 2018/01/02. 2002;180(1):51–60. Available from: https://www.cambridge.org/core/article/towards-a-standardised-brief-outcome-measure-psychometric-properties-and-utility-of-the-coreom/1A48F7373F484F7905D705C0774901D2. 10.1192/bjp.180.1.51 11772852

[26] Barkham M, Mellor-Clark J, Connell J, Cahill J. A core approach to practice-based evidence: A brief history of the origins and applications of the CORE-OM and CORE System. Couns Psychother Res [Internet]. 2006;6(1):3–15. Available from: 10.1080/14733140600581218.

[27] CORE Partnership. Is initial overall CORE-OM score an indicator of likely outcome? CORE Partnersh Occas Pap [Internet]. 2007;No 1. CORE. Available from: https://www.coreims.co.uk/site_downloads/OP1-initial_CORE-OM_score.pdf.

[28] Skre I, Friborg O, Elgarøy S, Evans C, Myklebust LH, Lillevoll K, et al. The factor structure and psychometric properties of the Clinical Outcomes in Routine Evaluation – Outcome Measure (CORE-OM) in Norwegian clinical and non-clinical samples. BMC Psychiatry [Internet]. 2013;13(1):99. Available from: 10.1186/1471-244X-13-99. 10.1186/1471-244X-13-99 PMC3618128 23521746

[29] Helsedirektoratet. Psykisk helse for voksne - ventetid [Internet]. 2019. Available from: https://www.helsedirektoratet.no/statistikk/kvalitetsindikatorer/psykisk-helse-for-voksne/gjennomsnittlig-ventetid-for-voksne-i-psykisk-helsevern.

[30] Bewick BM, McBride J, Barkham M. When clients and practitioners have differing views of risk: Benchmarks for improving assessment and practice. Couns Psychother Res [Internet]. 2006 Mar 1;6(1):50–9. Available from: 10.1080/14733140600581481.

[31] World Health Organization. Regional Office for South-East Asia. Equity in access to public health-report and documentation of the technical discussions. WHO Regional Office for South-East Asia. 2000. Available from: https://apps.who.int/iris/handle/10665/205027.

[32] Manser T, Foster S (2011). Effective handover communication: an overview of research and improvement efforts. Best Pract Res Clin Anaesthesiol.

[33] Helse-og omsorgsdepartementet. Lov om pasientrettigheter- Rett til helsehjelp. 1999. Available from: https://lovdata.no/LTI/lov/1999-07-02-63/§2-1.

[34] Helsedirektoratet. Metode ved utarbeiding av Helsedirektoratets prioriteringsveiledere. Prosjektdirektiv for Samarbeidsprojektet Riktigere prioritering i spesialisthelsetjenesten. 2018. Avaliable from: https://www.helsedirektoratet.no/veiledere/prioriteringsveiledere/aktuell-informasjon-om-lov-og-forskrift-for-prioriteringsveilederne/prosess-og-metode-ved-utarbeiding-av-prioriteringsveilederne/Metode%20ved%20utarbeiding%20av%20Helsedirektoratets%20prioriteringsveiledere.pdf/_/attachment/inline/632c602f-c5df-4fa9-ab27-37662d111e7a:118bea390bea65b1be00966d65ed507333ce69c6/Metode%20ved%20utarbeiding%20av%20Helsedirektoratets%20prioriteringsveiledere.pdf.

[35] Regionale komiteer for medisinsk og helsefaglig forskningsetikk. Eksempler på virksomhet som IKKE skal søke REK [Internet]. 2011. Available https://www.rekportalen.no/#hjem/s%C3%B8ke_REK.

