# Supplementary material for: Referral assessment and patient waiting time decisions in specialized mental healthcare: an exploratory study of early routine collection of PROM (LOVePROM)
Source: BMC Health Serv Res. 2022 Dec 20;22:1553. doi: 10.1186/s12913-022-08877-4 (PMC9764555; doi:10.1186/s12913-022-08877-4)
Supplement: Supplementary file 1 — Additional file 1: Table 1. Trigger levels of the Risk-to-others and Risk-to-self, based onquestions from the CORE-OM questionnaire. Table 2. The distribution of patients by CORE-OM categories. [file 12913_2022_8877_MOESM1_ESM.docx]

**Additional file**

**Supplementary Table 1.** Trigger levels of the Risk-to-others and Risk-to-self, based on questions from the CORE-OM questionnaire**.**

| Risk variable | CORE-OM Question | Trigger level |  | Levels |
| --- | --- | --- | --- | --- |
| Risk to others | Q6:  I have been physically violent to others | >1 |  | 0= Not at all;  1= Only occasionally;  2= Sometimes;  3= Often;  4= Most or all of the time |
|  | Q22:  I have threatened or intimidated another person | >2 |  |  |
| Risk to self | Q9:  I have thought of hurting myself | >2 |  | 0= Not at all;  1= Only occasionally;  2= Sometimes;  3= Often;  4= Most or all of the time |
|  | Q16:  I have made plans to end my life | >0 |  |  |
|  | Q24:  I have thought it would be better if I were dead | >2 |  |  |
|  | Q34:  I have hurt myself physically or taken dangerous risks with my health | >1 |  |  |

**Supplementary Table 2.** The distribution of patients by CORE-OM categories

|  | **n** | **Percentage (%)** |
| --- | --- | --- |
| **Below clinical cut-off** | 666 | 10.9 |
| **Mild** | 1374 | 22.5 |
| **Moderate** | 1969 | 32.2 |
| **Moderate severe** | 1452 | 23.8 |
| **Severe** | 647 | 10.6 |
